# Intestinal Microbiota Influences Non-intestinal Related Autoimmune Diseases

**DOI:** 10.3389/fmicb.2018.00432

**Published:** 2018-03-12

**Authors:** Maria C. Opazo, Elizabeth M. Ortega-Rocha, Irenice Coronado-Arrázola, Laura C. Bonifaz, Helene Boudin, Michel Neunlist, Susan M. Bueno, Alexis M. Kalergis, Claudia A. Riedel

**Affiliations:** ^1^Laboratorio de Biología Celular y Farmacología, Departamento de Ciencias Biológicas, Facultad de Ciencias Biológicas, Millennium Institute on Immunology and Immunotherapy, Universidad Andres Bello, Santiago, Chile; ^2^Facultad de Medicina, Millennium Institute on Immunology and Immunotherapy, Universidad Andres Bello, Santiago, Chile; ^3^Laboratorio de Inmunobiología, Facultad de Medicina, Departamento de Biología Celular y Tisular, Universidad Nacional Autónoma de México, Mexico City, Mexico; ^4^Departamento de Genética Molecular y Microbiología, Facultad de Ciencias Biológicas, Millennium Institute on Immunology and Immunotherapy, Pontificia Universidad Católica de Chile, Santiago, Chile; ^5^Unidad de Investigación Médica en Inmunoquímica Hospital de Especialidades Centro Médico Nacional Siglo XXI, Instituto Mexicano del Seguro Social, Mexico City, Mexico; ^6^Institut National de la Santé et de la Recherche Médicale U1235, Institut des Maladies de l'Appareil Digestif, Université de Nantes, Nantes, France; ^7^Departamento de Endocrinología, Facultad de Medicina, Pontificia Universidad, Metropolitana, Chile

**Keywords:** microbiota, autoimmune disease, gut, microbiome, skin, CNS

## Abstract

The human body is colonized by millions of microorganisms named microbiota that interact with our tissues in a cooperative and non-pathogenic manner. These microorganisms are present in the skin, gut, nasal, oral cavities, and genital tract. In fact, it has been described that the microbiota contributes to balancing the immune system to maintain host homeostasis. The gut is a vital organ where microbiota can influence and determine the function of cells of the immune system and contributes to preserve the wellbeing of the individual. Several articles have emphasized the connection between intestinal autoimmune diseases, such as Crohn's disease with dysbiosis or an imbalance in the microbiota composition in the gut. However, little is known about the role of the microbiota in autoimmune pathologies affecting other tissues than the intestine. This article focuses on what is known about the role that gut microbiota can play in the pathogenesis of non-intestinal autoimmune diseases, such as Grave's diseases, multiple sclerosis, type-1 diabetes, systemic lupus erythematosus, psoriasis, schizophrenia, and autism spectrum disorders. Furthermore, we discuss as to how metabolites derived from bacteria could be used as potential therapies for non-intestinal autoimmune diseases.

## Introduction

Our body is colonized by millions of microorganisms that can survive in extreme environments surpassing difficult conditions, such as low pH or low oxygen (Peterson et al., 2015). The skin, gut, nasal, and oral cavities and genital tract are colonized by hundreds of different types of microorganisms and are known as “normal flora” or microbiota (Peterson et al., 2015). Lederberg defined the microbiota in 2001 as “*the ecological community of commensal, symbiotic, and pathogenic microorganisms that share our body space”* (Lederberg, 2001). For some authors the concept of microbiota comprehends mainly bacteria and while the concept of microbiota comprehends several different species among them are bacteria, archaea, fungi and viruses (Selber-Hnatiw et al., 2017). Recent scientific advances supported additionally by “*omics analyses”* have been crucial for the generation of a large amount of data relative to the composition of the microbiota (Almonacid et al., 2017). In fact, scientific progress has allowed the identification of the composition of the microbiota and the identification of specific microorganisms that live in the gut (Ferreira et al., 2017). The analysis of this information has contributed to revealing the complex relationship between the microbiota and the host. Evidence in the literature has shown that alterations in the proportion of these microorganisms can be associated to pathologies affecting humans (Aarts et al., 2017; Almonacid et al., 2017; Ferreira et al., 2017). Along these lines, in the past few years several scientific publications have shown a possible association between microbiota alterations and autoimmune diseases (Alkanani et al., 2015; Ma et al., 2015; Miyake et al., 2015; Breban et al., 2017; Kohling et al., 2017). These pathologies are characterized by an immune response against the body's own tissues causing inflammation and destruction of tissues and/or organs (Nagy et al., 2015). Autoimmune diseases are especially frequent in western countries, affecting majorly women (Davidson and Diamond, 2001). It has been proposed that lifestyle in this “modern era” can be affecting the microbiota composition causing a deregulation of the immune system (Berbers et al., 2017). Evidences in the literature have shown a strong link between microbiota composition and intestinal autoimmune diseases, such as Crohn‘s disease and inflammatory bowel disease (IBD) (Matsuoka and Kanai, 2015; Nishida et al., 2017; Powell and MacDonald, 2017). However, host gut microbiota seems also capable of influence autoimmune diseases that target tissues other than the intestine, including Type 1 diabetes (De Groot et al., 2017), multiple sclerosis (Hindson, 2017), arthritis (Felix et al., 2017), and psoriasis (Yan et al., 2017). Interestingly diseases like schizophrenia and autism are now considered to also have an inflammatory component suggesting that these ailments could also be associated to changes in intestinal microbiota (Dickerson et al., 2017; Vasquez, 2017; Wu, 2017; Yang et al., 2017; Cox and Weiner, 2018; Kopec et al., 2018). The aim of this review article is to analyze recent information supporting an association between gut microbiota composition and non-intestinal autoimmune diseases.

## The microbiota through human life

An adult of 70 kg in average has around 39 trillion of bacteria and 30 trillion of human cells (Sender et al., 2016) and at least 20% of the metabolites in the blood are derived from commensal bacteria (Rook et al., 2017). The gut microbiota consists of about 2,000 different bacterial species (Llorente and Schnabl, 2015) and most of them reside at the distal intestine (Kamada et al., 2013b). In general, human gut microbiota is comprised by two main dominants phyla Firmicutes and Bacteroidetes, which are susceptible to alterations due to factors such as age, genetics, diet, environment, and infection (Gill et al., 2006). The neonatal microbiota is highly different compared to adult microbiota (Pickard et al., 2017). Neonatal microbiota is strongly influenced by type of delivery at birth. Thus, a vaginal delivery allows the colonization of the mother's gastrointestinal microorganisms to the neonate, meanwhile in a C-section delivery the infant will present more microorganisms related to mother's skin (Wampach et al., 2017).

Certain data support the notion that bacteria colonization in humans will begin in the gestation at the womb (Stinson et al., 2017). Consistently, several reports have detected the presence of bacterial DNA in the amniotic fluid, umbilical cord, placenta, meconium, and fetal membranes (Khan et al., 2016; Stinson et al., 2017; Tschoeke et al., 2017). This evidence is in contrast with the hypothesis of the sterile womb and that bacteria colonization in humans begins only at birth or at breastfeeding (Funkhouser and Bordenstein, 2013). The neonatal microbiota initially will resemble very much to individual maternal microbiota (Wampach et al., 2017). During the following years, the microbiota will be shaped and changed by nutritional, physiological, and/or pathological events occurring through life (Chu et al., 2016).

Evidence suggests that a proper microbiota homeostasis is required for the maturation of central nervous system (CNS), as well as the immune system during different developmental stages, such as infancy, adolescence, and adulthood (Rook et al., 2017). Certain microorganisms included in the microbiota will contribute to an appropriate development of various human tissues and organs. However, other microorganism could increase the susceptibility to suffering certain pathologies (Berbers et al., 2017). Therefore, the diet is an important factor that can lead to changes in the microbiota composition (Cui et al., 2017). It has been reported that only 24 h are sufficient to alter the composition of the microbiota after a change in the diet of an individual (Wu et al., 2011). For example, high fat diets increase the presence of enterotypes, such as *Bacteroides* meanwhile a fiber-rich diet increases the amount of the *Prevotella* genus (Wu et al., 2011). It has been shown that inappropriate changes in the microbiota composition, known as dysbiosis, could cause harmful consequences to the host (La Fata et al., 2017). For example dysbiosis has been reported for patients suffering from type I and type II diabetes, IBD and colorectal cancer (CRC) (Peterson et al., 2015). Because it would be of importance to understand how the microbiota composition can impair the wellness of the host, significant research efforts are currently in place to develop new treatments for these pathologies based on restoring a normal microbiota composition.

## The intestinal barriers for the microbiota

The gut has developed different mechanisms to ensure a beneficial intestinal microbiota composition, as well as for regulating microbiota overgrowth and restricting pathogen colonization (Llorente and Schnabl, 2015; Gensollen et al., 2016). It is thought that the gut can produce an intestinal barrier by secreting mucus and pro-inflammatory molecules that contribute to the establishment of innate and adaptive immunity (Feng and Elson, 2011).

Intestinal epithelial cells are the first constituents of the gut barrier and they cover the intestinal lumen and separate the gut microbiota from the immune system (Farhadi et al., 2003; Feng and Elson, 2011). Epithelial cells are maintained together by tight junctions (TJs), adherents junctions (AJ), and desmosomes (Hartsock and Nelson, 2008). TJs are multiprotein complexes comprised of integral membrane proteins, such as claudins, occludins, and junctional adhesion molecules (Hartsock and Nelson, 2008). The TJs regulate the passing of solutes and fluids through the epithelial cells by passive paracellular diffusion (Choi et al., 2017). As part of the epithelial cell barrier are goblet cells, which secrete glycoproteins to the lumen forming an inner mucus layer that is closer to epithelium and an outer mucus layer that is in contact with bacteria (Hooper and Macpherson, 2010). Additionally, epithelial cells can secrete antimicrobial proteins, such as defensins, cathelicidins, and C-type lectins (Chairatana and Nolan, 2017). These molecules contribute at controlling bacterial growth by either enzymatically degrading their cell wall, disrupting the inner membrane or depriving bacteria from essential heavy metals (Mukherjee and Hooper, 2015). Additionally, enterocytes, enteroendocrine cells, globet cells, and Paneth cells can also produce antimicrobial proteins contributing to the antimicrobial activity (Chairatana and Nolan, 2017).

The three main lymphoid structures of gut immune system that locate at the mucosa are: (1) the Peyer's patches (PP), which is the mucosa-associated lymphoid tissue that can be found in clusters; (2) the lamina propria (LP) located as an isolated lymphoid tissue where effector lymphocytes secrete cytokines and immunoglobulins; and (3) the epithelium layer in which intraepithelial resident lymphocytes can be found (Richards et al., 2016; Shi et al., 2017). The secretion of IgA is considered to be an antimicrobial agent that is accomplished by the help of dendritic cells (DC) from the PP. IgA interacts with bacteria impeding their adhesion to epithelial cells and inhibiting bacterial motility (McGuckin et al., 2011).

The gut microbiota is tightly associated and has constant communication with the mucosal immune system. It is thought that the mucosal immune system limits the invasion of tissues by the normal flora, which entails a high microbial diversity as well as potential pathogens that could have been ingested with the diet (Hooper and Macpherson, 2010). Such a function is in part carried out by DCs located at the mucosal surface where they uptake antigens and prime lymphocytes. DCs can directly sample normal flora and pathogenic bacteria (Kelsall, 2008). Despite all of these barrier mechanisms, bacteria can find ways for trespassing them and go across the epithelial layer triggering bacteria killing mechanisms. Rapidly, trespassing bacteria suffer phagocytosis and elimination by the LP macrophages (Kelsall, 2008).

**Figure 1:**
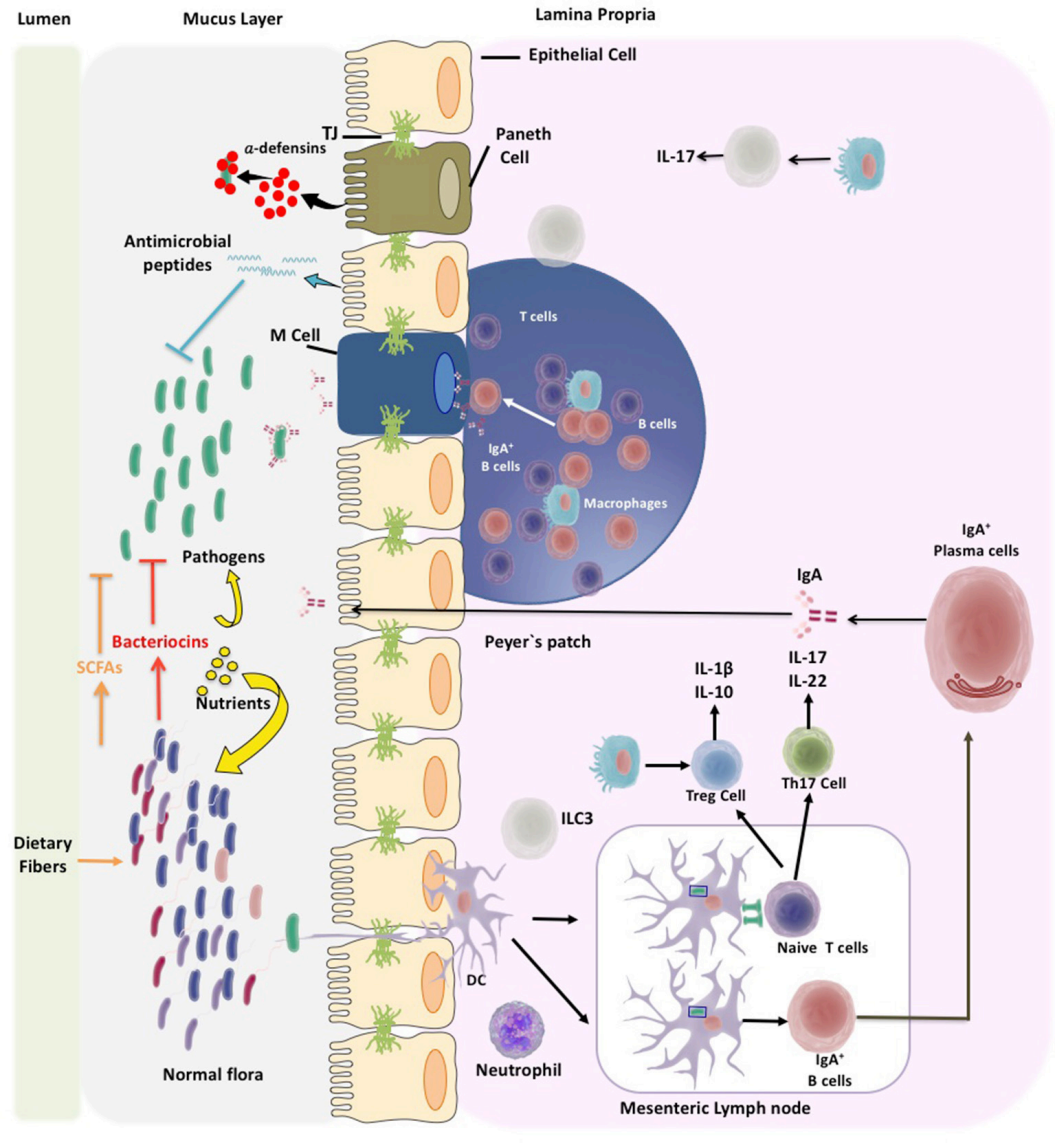
Microbiota and pathogen control mechanisms of the gut immune system. The immune system of the gut has to deal with wide diversity of microbiota and pathogens. Thus, the immune system will help to establish a beneficial microbiota composition at the gut. Different mechanisms of the gut immune system have been discovered that control microbiota overgrowth and pathogens invasion. The gut immune system locates in the mucosa layer of the intestine mainly at the epithelium layer and Peyer's patches at the lamina propia. Enterocytes, enteroendocrine cells, globet cells, and Paneth cells, located in the intestinal epithelial layer, secrete antimicrobial peptides like defensins. It has been shown that defensins are produced in the course of innate immune defense to activate the adaptive immune response. Another mechanism to control microbiota is the secretion of IgA which is accomplished by dendritic cells (DC). IgGA by interacting with bacteria impedes their adhesion and inhibits bacteria motility. DCs are localized at mucosal surfaces in antigen uptake sites and at inductive lymphoid tissue; they can directly sampling the normal flora and pathogenic bacteria. In the mesenteric lymph node DCs can promote differentiation of T cells, to regulate immune tolerance. Once T cells are activated they differentiate to T helper cells (Th), like Th1, Th2, or Th17 cells. Intestinal DCs will also regulate the differentiation of T cells to T regulatory (Treg) cells. Treg cells have the capacity to suppress the activation and proliferation of Th cells by the secretion of anti-inflammatory cytokines.

Activated DCs can promote the differentiation of T cells to regulate immune tolerance (Shi et al., 2017). In fact, there is a high content of T cells at the intestinal mucosa that can be divided in two major subpopulations known as type A or conventional (TCRαβ) located at the PPs and mesenteric lymph nodes and type B or non-conventional (TCRγδ) that can be found almost exclusively at the epithelium (Van Wijk and Cheroutre, 2010). Naïve T cells become activated into effector helper T cells (Th) by mainly differentiating into: (1) Th1 cells that contribute to the elimination of intracellular pathogens; (2) Th2 cells that protect against parasites and mediate allergic reactions; or (3) Th17 cells that contribute to the clearance of foreign pathogens (Geremia et al., 2014). Intestinal DCs can also regulate the differentiation of T cells to regulatory T cells (Tregs). The role of Tregs is to reduce and control the immune response in part by suppressing the activation and proliferation of T helper cells through the secretion of anti-inflammatory cytokines (O'garra and Vieira, 2004). Several studies support the notion that an alteration of the balance between T helper and Treg cells can be closely associated to intestinal autoimmune pathologies (Fasching et al., 2017; Figure 1).

## Modulation of T cell differentiation by the microbiota

Microbiota members can regulate the immune response through secretion of metabolites, such as short-chain fatty acids (SCFA). SCFAs are produced in the colon by the microbiota through fermentation of non-digestible carbohydrates including cellulose or inulin among others (Chen et al., 2015). The main products are acetate, propionate and butyrate, which are absorbed by the colon (Rios-Covian et al., 2016). While butyrate acts as an energy source for epithelial cells (Jung et al., 2015) and facilitates tight junction assembly (Peng et al., 2009), acetate and propionate are substrates for gluconeogenesis and lipogenesis in the liver and other peripheral organs (Rios-Covian et al., 2016).

SCFAs can modulate the intestinal immune response (Tremaroli and Backhed, 2012) by regulating T cell differentiation (Cavaglieri et al., 2003), epithelial barrier function, production of antimicrobial peptides, and the secretion of pro-inflammatory mediators (Johnson-Henry et al., 2014). Administration of butyrate in an animal model of colorectal colitis ameliorates the symptoms by increasing the percentage of Tregs and the production of IL-10 and IL-12 in peripheral blood, with an concomitant decrease of RORγt (a Th17 biomarker), IL-17 and IL-23 levels (Zhang et al., 2016). Furthermore, the addition *in vitro* of butyrate to human peripheral blood mononuclear cells (PBMCs) increased the differentiation of Tregs suggesting for this molecule a regulatory role in Treg/Th17 balance that influences the immune response (Zhang et al., 2016). Administration of SCFAs has been used in animal models of experimental autoimmune encephalomyelitis (EAE). It was observed that the oral administration of SCFAs butyrate, acetate, and propionate could significantly decrease EAE clinical score in mice (Mizuno et al., 2017). In these experiments, propionate showed the higher capacity to protect animals from the development of EAE (Mizuno et al., 2017) Interestingly, while treatment with propionate increased the frequency of Tregs in lymph nodes, treatment with butyrate did so in the spleens (Mizuno et al., 2017). These results reinforce the notion that SCFAs from the intestine can regulate systemic inflammation that is mediated by lymphocytes.

Hashimoto et al. by using *Ace2* knockout mice showed that a protein-free diet alters intestinal immunity (Hashimoto et al., 2012). Angiotensin converting enzyme-2 (ACE2) is a key regulatory enzyme of the renin-angiotensin system (RAS) as it catalyzes the conversion of angiotensin II (Ang II) to angiotensin 1–7 (Ang 1–7) the latter can bind the G-coupled protein Mas receptor inducing vasodilatation contrasting the effects of the binding of Ang II to its receptor AT-1 that promotes vasoconstriction and hypertension (Perlot and Penninger, 2013). ACE2 knockout male mice or Ace2^−/y^ mice (Ace deficient at the × chromosome) induced with colitis and protein-deprived showed increased infiltration, ulceration, weight loss and higher diarrhea scores, as well as decreased serum levels of tryptophan (Trp) (Hashimoto et al., 2012). Trp is an essential amino acid for mammals and can only be obtained through the diet (Badawy, 2017). These knockout mice supplemented with Trp significantly improved colitis symptoms (Hashimoto et al., 2012). Additional analyses showed that these mice display an altered gut microbiota composition and an increase production of antimicrobial peptides, as compared to Trp-supplemented mice (Hashimoto et al., 2012).

Moreover, it has been observed that the microbiota produces catabolites from Trp or Trp-indole derivatives, such as Indole 3-acetamide, indole-3-acetic acid and indole-3-lactic acid and modulates the mucosal immune response through IL-22, which is produced by innate lymphoid cells 3 (ILC3) (Zelante et al., 2013). Consistently with these findings, Lamas et al. showed that the colitis associated knockout mice for *Card9* display a microbiota dysbiosis (Lamas et al., 2016). An adaptor protein involved in the immune response against fungi dysbiosis (Etienne-Mesmin et al., 2017). Authors showed that *Card9* knockout mice missed a Trp-metabolizing bacterium. Thus, the consequence is that these mice had low content of indole derivatives, which are important for the production of IL-22 by the ILC3 and T cells at the mucosa. Low levels of IL-22 generate a pro-inflammatory environment (Lamas et al., 2016), because this cytokine has anti-inflammatory properties and belongs to the IL-10 cytokine family (Parks et al., 2015). IL-22 participates in host defense against extracellular pathogens by eliciting innate defensive mechanisms that promote the expression of antimicrobial peptides at mucosal surfaces (Rutz et al., 2013) and is also involved in tissue repair by enhancing epithelial cell proliferation (Aujla and Kolls, 2009).

Additionally, IL-22 can influence intestinal epithelial cell glycosylation by inducing the expression of fucosyltransferase 2 (Fut2) that catalyzes the fucosylation of membrane proteins, a post translational modification needed for protection against enteric pathogens, such as *S. typhimurium* (Okumura and Takeda, 2017). Recent data support the notion that microbiota has immune-modulatory properties, however little is known about the identification of the specific bacteria genera responsible for the phenotype and also few is known at molecular level for which mechanisms these bacteria modulate the inflammatory state of the intestine. So far, it has been described that the presence of polysaccharide A (PSA) in the gut commensal *Bacteroides fragilis* induces the secretion of IL-10 by Tregs, which in turn decreases inflammation in the gut and in distant tissues, such as the brain (Ochoa-Reparaz et al., 2010; Dasgupta et al., 2014). These findings highlight the role of metabolites produced by the intestinal microbiota to modulate inflammation and their potential use as a therapeutic tool to treat inflammatory and autoimmune diseases.

## What do we know about the role of the microbiota in non-intestinal autoimmune diseases?

### Autoimmune diseases

Autoimmune diseases are pathologies characterized by an inappropriate immune response against own tissues and molecules that results in tissue-specific or systemic inflammation that leads to organ damage and malfunction (Rose and Bona, 1993; Marmont, 1994). Causes for autoimmune disease are multifactorial and range from genetic predisposition to the exposition of environmental agents, such as infectious agents, xenobiotics, drugs, or stress (Davidson and Diamond, 2001). The disease progresses from initial naive lymphocyte activation to a chronic state characterized by an increase in the number of autoantigens targeted by T cells and antibodies. Activated autoreactive B cells can function as antigen presenting cells for novel peptides and express co-stimulatory molecules. Antigens are processed and presented to naive T cells leading to the activation of additional autoreactive B cells that present new epitopes up to a point in which there is autoreactivity to a large number of autoantigens (Lanzavecchia, 1995; Liang and Mamula, 2000). The production of autoantibodies induces damages to tissues by the formation of immune complexes, cytolysis, or phagocytosis of target self-cells and interfering with proper tissue and cellular functions (O'Garra et al., 1997). Although there are several autoimmune diseases, in this review we will focus on non-intestinal autoimmune disorders, for intestinal autoimmune diseases, please refer to other reports (Gallo et al., 2016; Blander et al., 2017; Passos and Moraes-Filho, 2017).

### Graves's disease and hashimoto's thyroiditis

The Grave's disease (GD) is an autoimmune disease characterized by the targeting of antigens derived from the thyroid gland. In GD there are autoantibodies against the thyroid stimulating hormone receptor (TSHR) (Kristensen, 2016). These autoantibodies activate the TSHR inducing the synthesis and secretion of thyroid hormones by the thyroid gland and causing hyperthyroidism. GD is the most common cause of hyperthyroidism and is more frequently observed in women than in men (Pokhrel and Bhusal, 2017). Shor et al. evaluated the prevalence of gastrointestinal auto antibodies in patients with Hashimoto's thyroiditis and Grave's disease (Shor et al., 2012). These are anti-gliadin antibodies (AGA), tissue transglutaminase (tTG) and anti *Saccharomyces cerevisiae* antibodies (ASCA). In particular ASCA have proven to be sensitive and highly specific for Crohn's disease. ASCA antibodies were highly prevalent in patients with GD (Shor et al., 2012). Analysis of fecal samples from GD patients showed an increased content of yeast supporting Schor's analyses (Covelli and Ludgate, 2017). It has also been observed the presence of antibodies against *Yersinia enterocolitica* and to *Helicobacter pylori*, but these responses vary among patients and are not observed in all the analyzed patients (Kohling et al., 2017). Next-generation sequencing projects intended to analyze and identify bacteria species in patients with GD. Using a TSHR immunized mouse model, it was observed an alteration of immunized animals when compared to controls. In humans, this modification is not fully observed, so far it has been observed in a small number of patients a significant decrease of the *Bacteroides* genus (Indigo, 2017). These are the first reports associating the gut microbiota and GD; therefore additional work must be accomplished to better understand the influence of the gut microbiota on the development of GD.

Hashimoto's thyroiditis (HT) is an autoimmune disease that is characterized by the infiltration of mononuclear cells in the thyroid, together with the production of autoantibodies against thyroglobulin and thyroid peroxidase (TPO) (Antonelli et al., 2015). It is thought that environmental factors, such as diets higher in iodide, contribute to the etiology of HT (Rose et al., 2002). Recently, research efforts have focused on the involvement of microbiota in the pathogenesis of autoimmune diseases. The transfer of microbiota from conventional rats to specific pathogen free (SPF) rats increased the susceptibility of the latter to experimental autoimmune thyroiditis (Penhale and Young, 1988), which provides further support for an influence of the microbiota during HT pathogenesis. The use of the probiotic mixture (VSL#3™) has been successful to reduce the susceptibility to developing autoimmune diseases, such as Type 1 diabetes and colitis by enhancing the production of IL-10 in Peyer's patches and the spleen (Calcinaro et al., 2005; Di Giacinto et al., 2005). Along these lines, it was important to explore whether probiotics could have a positive effect on HT. Contrarily to what was initially thought, the use probiotic strains of *Lactobacillus rhamnosus* HN001, *Bifidobacterium lactis* HN019, and *L. rhamnosus* GG failed to improve the disease outcome in a mouse model for autoimmune thyroiditis (Zhou and Gill, 2005). It has been demonstrated that a dysbiosis state can alter the epithelial barrier permeability leading to a condition known as “leaky gut” (Vaarala et al., 2008). At the histologic level, this is observed as morphological changes in epithelial cells and lymphocyte infiltration (Fritscher-Ravens et al., 2014). Interestingly, a similar observation has been made in patients with HT, in which both the space of two adjacent microvilli and the microvilli thickness are significantly increased. Furthermore, these patients were also evaluated for functional mucosal alterations using a lactulose/manitol test showing an increase in the recovery of lactulose/mannitol, which is consistent with the histological observations (Sasso et al., 2004). These data suggest that the microbiota and the epithelial barrier play an important role of during the pathogenesis of HT.

### Type I diabetes

Type 1 diabetes (T1D) is the most prevalent autoimmune disease in young people (<20 years), with a peak at 10–14 years old (Maahs et al., 2010) being more common in boys than in girls (Atkinson et al., 2014). The incidence of T1D is very variable worldwide, being higher in Europeans countries probably due to environmental factors (Xie et al., 2014) T1D is characterized by a T cell-mediated (CD4^+^ and CD8^+^) destruction of β pancreatic cells (Atkinson et al., 2014). There are three major auto antigens associated to T1D, which are insulin, GAD65 (glutamic acid descarboxylase, 65 kDa isoform), and IA2 (Insulin autoantigen 2) (Ounissi-Benkalha and Polychronakos, 2008). The presence of antibodies specific for these antigens has been observed in the serum of T1D patients (Miao et al., 2007). A classical trio of symptoms characterizes T1D, which are polydipsia, polyphagia, and polyuria, accompanied by an overt hypoglycemia (Atkinson et al., 2014). All of them are used as hallmarks for T1D diagnosis in high-risk individuals, such as children and adolescents (Leslie, 2010).

The 60% of patients suffering from T1D has been attributed to a genetic cause (Ounissi-Benkalha and Polychronakos, 2008). The 50% of heritability of T1D is attributed to the human leukocyte antigen (HLA) alleles located in chromosome 6 and the rest is to non-HLA loci (Barrett et al., 2009; Redondo et al., 2017). It has been reported that are at least 40 non-HLA loci such *INS, CTLA4, PTPN22*, and *IL2RA* can contribute to disease susceptibility (Barrett et al., 2009). Moreover, T1D can also be triggered by environmental factors, such as cesarean or vaginal birth, diet, early infections in life etc. (Rewers and Ludvigsson, 2016). Experimental evidence has shown that the intestinal microbiota could induce T1D by priming the immune system at an early postnatal period (Endesfelder et al., 2016).

The first evidence linking the gut immune system and T1D derives from animal models. Non-obese diabetic (NOD) mice fed with regular commercial cereal-based chow and mice fed with a 10% casein-based diet presented the highest rates of T1D among the experimental groups (Elliott et al., 1988). These rates (26.9% cereal-based chow vs. 19.1% casein-rich diet) were significantly higher than the expected for this type of mice, which normally develop insulin-dependent diabetes at 200–250 days of age. Authors observed that the incidence of T1D in these mice was due to the 10% of casein in this diet, interestingly this percentage corresponds to the percentage of casein in cow milk (Elliott et al., 1988). Another important observation in children suffering T1D was the presence of antibodies against bovine serum albumin, a protein also contained in cow milk (Savilahti et al., 1988; Karjalainen et al., 1992; Saukkonen et al., 1996). These two observations support the notion that the diet could trigger the development of T1D, due that it contains potential antigens that will prime the immune system (Mejia-Leon and Barca, 2015; Rewers and Ludvigsson, 2016; Virtanen, 2016).

The notion that antigens derived from the diet can prime the immune system, suggests that the immune system is in contact with antigens and the intestinal permeability must be altered. In fact, there is evidence supporting a relationship between T1D and high intestinal permeability (Vaarala, 2008; Li and Atkinson, 2015; Maffeis et al., 2016). A study performed in 46 non-celiac T1D patients showed a significant increase of intestinal permeability as compared to healthy controls (Secondulfo et al., 2004). Authors performed electronic transmission microscopy (TEM) analyses over intestinal biopsies from non-celiac T1D patients. They observed a partial decrease in the microvilli together with morphological alterations at the tight junction domains (Secondulfo et al., 2004). Another study showed that T1D patients have high intestinal permeability measured as the urine levels of lactulose and mannitol 5 h after ingestion and high levels of zonulin in the serum (Sapone et al., 2006). Zonulin is a protein that can regulate intestinal permeability by disassembling tight junctions (Fasano et al., 2000). Studies performed in Biobreeding diabetes-prone (BBdp) rats, widely used as an animal model for studying human T1D (Bortell and Yang, 2012), showed increased intestinal permeability (Meddings et al., 1999). Using this animal model Neu et al. found in the small intestine of these animals a high number of globet cells and high intestinal mucus secretion before the onset of the disease, reflecting an inflammatory response at the intestine (Neu et al., 2005).

Other studies have found that intestinal microbiota could increase the probability to develop T1D. Studies performed in non-obese diabetic (NOD) mice at young age, when these animals are prediabetic, were infected by an oral gavage with wild type *C. rodentium* showed higher intestinal permeability, developed earlier insulitis (Lee et al., 2010) and showed an increased lymphocytic infiltration at the pancreas Langerhan's islets (In't Veld, 2011). These authors showed that the mutant strain of *C. rodentium* that lacks the ability to disrupt the intestinal barrier was unable to induce insulitis (Lee et al., 2010). Maffeis et al. observed in Italian T1D affected children an increased intestinal permeability that correlates with alterations in the microbiota composition (Maffeis et al., 2016). Interestingly, the authors found three microbial markers (*D. invisus, G. sanguinis*, and *B. longum*) highly represented in T1D affected children as compared to healthy controls (Maffeis et al., 2016). Furthermore, it has been observed that bio-breeding diabetes-resistant (BBDR) rats present more probiotic bacteria, such as *Bacterioides, Eubacterium*, and *Ruminococcus* (Roesch et al., 2009). It has also been observed in humans that the *Bacteroidaceae* family is over-represented in children with T1D together with a decrease of intestinal microbiota dominant species as *Bifidobacterium adolescentis* and *B. pseudocatenulatum* (De Goffau et al., 2013). Microbiota composition can be influenced by age, with major changes observed at early ages (Koenig et al., 2011; Arrieta et al., 2014; Rodriguez et al., 2015). Kostic et al. described that the microbiota of genetically-predisposed infants from 3 to 36 months old, based on HLA genotyping, has reduced alpha diversity and overabundance of *Blautia, Rikenellaceae, Ruminococcus*, and *Streptococcus* genera (Kostic et al., 2015). In contrast, Maffeis et al. (2016) showed that *D. invisus* was completely absent in this samples indicating that there is still contradictory data regarding the microbiota composition in T1D patients.

### Multiple sclerosis

Multiple sclerosis (MS) is the most common autoimmune disease that has the CNS as a target (Reich et al., 2018). The immune system in the MS patients reacts against proteins that are found in myelin and neurons inducing axonal damage and neuronal death (Lemus et al., 2018). The MS patient develops several symptoms that lead to chronic disability including cognition impairment, loss of motor control, and sensitivity (Yong et al., 2017). Several reports have shown that MS patients have dysbiosis at the intestinal microbiota and it has been proposed that MS patients could have a specific type of microbiome (Chen et al., 2016; Probstel and Baranzini, 2017; Shahi et al., 2017; Tremlett and Waubant, 2017). It was shown that MS patients have a relatively low abundance of the *Bacterioides, Parabacteroides, Prevotella*, and *Lactobacillus* genera and an increased abundance of *Akkermansia, Blautia, Ruminococcus*, and *Bifidobacterium* (Jangi et al., 2016; Freedman et al., 2017). Interestingly, *Akkermansia* is a mucin-degrading microorganism that transforms mucin into SCFAs, suggesting that this microorganism might be trying to compensate the inflammatory state of the MS patient (Derrien et al., 2004, 2011). Cekanaviciute et al. found that samples of gut microbiome from MS patients impaired the differentiation of T cells to CD25^+^FoxP3^+^ Treg cells (Cekanaviciute et al., 2017). These authors found that MS patients have a high presence of the *Akkermansia* genus, specifically *A. calcoaceticus* and *A. muciniphila* (Cekanaviciute et al., 2017). Further, it was observed that PBMCs from healthy donors showed an increased differentiation into effector T cells as compared to Treg cells, when exposed to *A. calcoaceticus* extracts from MS patients (Cekanaviciute et al., 2017). Once PBMCs are exposed to *A. muciniphila* extracts from MS patients the differentiation of T cells inclined toward to Th1 cells, suggesting that the microbiota of MS patients has a pro-inflammatory effect (Cekanaviciute et al., 2017).

There are several animal models to study MS, one of them is the experimental autoimmune encephalomyelitis (EAE) (Robinson et al., 2014). EAE has the advantage of reproducing most of the symptoms observed in humans and disease is mediated by T cells, as it is the case for MS patients (Stromnes and Goverman, 2006). The induction of EAE in rodents generates a T cell-mediated response, mainly of Th1 and Th17 types (Kleinewietfeld and Hafler, 2013). Interestingly, the function these cells is influenced by the composition microbiota (Wu and Wu, 2012). It has been shown that in normal conditions commensal microorganisms, such as segmented filamentous bacteria can activate these cells to keep a healthy immune response (Ivanov et al., 2009). Noteworthy, Lee et al. showed that the germ-free mice were more resistant to EAE (Lee et al., 2011). The authors observed that some of these mice did not develop symptoms, while and developed only mild symptoms and had a shorter period of EAE (Lee et al., 2011). Experiments of microbiota transfer from specific pathogen free mice (SPF) mice to germ-free mice enhanced the EAE symptoms in germ-free mice, supporting the notion that microbiota can influence the immune response during EAE (Lee et al., 2011).

Other studies have found that the microbiota can alter the ratio of cells that play important roles during autoimmune diseases, such as effector T cells vs. Tregs (Molloy et al., 2012; Kosiewicz et al., 2014). For example, *in vitro* re-stimulation with MOG_35−55_ of T cells from germ-free mice and SPF mice that have been previously immunized with MOG/CFA showed in SPF mice an increased secretion of IFNγ and IL-17, as compared to germ-free mice (Lee et al., 2011). Germ-free mice have increased frequencies of Tregs at day 8th before the onset of EAE and at day 15 at the peak of EAE symptoms (Lee et al., 2011). Recently, it was reported that EAE could be induced in mice that received microbiota from MS patients (Berer et al., 2017). By using next-generation sequencing analyses it was shown that the microbiota composition of MS patients has a low content of the *Sutterella* genus (Berer et al., 2017). It has been observed that this bacterium plays a beneficial role in patients that suffer from IBD (Morgan and Harris, 2015). There is evidence that Bacteroides can be beneficial for maintaining the homeostasis in models of intra-abdominal sepsis and experimental colitis (Tzianabos et al., 1995; Pagliuca et al., 2016). This seems to be the case for *B. fragilis*, as its polysaccharide (PSA) induces IL-10 secretion by Tregs (Ochoa-Reparaz et al., 2010). In fact, oral treatment with purified *Bacterioides fragilis* PSA has been shown to be beneficial because it reduces EAE scores, demyelination, IFNγ, and IL-17 and increases T cells conversion to Tregs and the content of IL-10 (Ochoa-Reparaz et al., 2010). Oral immunization with an oral vaccine of attenuated *Salmonella typhimurium* expressing the colonization factor antigen I (CFA/I) of Enterotoxigenic *Escherichia coli* (ETEC) to mice that suffer EAE decreased the EAE scores showing a decreased infiltration of TCD4^+^ cells in the spinal cord (Jun et al., 2005). CFA/I plays an important role in the attachment of ETEC to the intestinal epithelia an important step for diarrhea pathogenesis (Li et al., 2009).

The question as to how the microbiota modulates de immune response in the MS and/or EAE remains to be addressed. Rumah et al. showed that bacteria metabolites could modulate the immune response (Rumah et al., 2013). These authors isolated a strain of *Costridium perfringens* type B bacillus from a woman that suffered MS and showed ovoid lesions at the *corpus callosum* (Rumah et al., 2013). By using a mouse model it was shown that protoxin (a protein secreted by this strain) caused BBB disruption (Finnie, 1984) and binds to myelin in the CNS inducing oligodedrocyte death, as observed in frozen sections of mouse retina (Rumah et al., 2013). Farrokhi et al. found that serum from MS patients has low levels of the lipid 654, this lipopeptide is secreted by commensal intestinal *Bacteroidetes* in healthy people (Farrokhi et al., 2013). It has been reported that the lipid 654 could work as a ligand for the human toll-like receptor 2 (TLR2), thus it could contribute at down-regulating innate immunity (Clark et al., 2013). The work of Farrokhi suggests that low levels of lipid 654 could be used as a biomarker of MS, however further research is required to demonstrate that this diagnostic approach would be accurate.

Evidence derived from the EAE model suggests that SCFAs can improve and balance the immune response. Mizuno et al. observed that the oral administration of propionate decreased EAE clinical scores and increased Tregs at the lymph nodes, thus it could favor the suppression effect of Tregs (Mizuno et al., 2017). Additional interesting work has provided evidence that supports the notion that the first symptoms of EAE begins at the intestine (Nouri et al., 2014). Nouri et al. in their article showed that there is an unbalance of Treg/Th17 toward Th17 pro-inflammatory response at the intestine (Nouri et al., 2014) Authors also showed an increased intestinal permeability, altered intestinal morphology across the small intestine characterized by depth crypt, gross mucosal thickness, and high expression of zonulin (Nouri et al., 2014). Summarizing, the evidence available in the literature suggests that intestinal microbiota could potentiate or modulate the immune response of MS or EAE specifically the ration or balance of Treg/Th17.

### Systemic lupus erythematosus

Systemic Lupus Erythematosus (SLE) is a systemic autoimmune disease with unknown etiology that affects predominantly women. SLE is characterized by the presence of hyperactive and aberrant antibody response to nuclear and cytoplasmic antigens (La Paglia et al., 2017). Regarding the influence of microbiota over SLE development, microbiota composition analyses showed that SLE patients display intestinal dysbiosis. A decrease in the *Firmicutes* with an increase of the *Bacteroides* phyla was observed (Hevia et al., 2014). These bacteria are the most abundant components of the human microbiota and the same pattern has been observed in patients with Crohn's disease (Qin et al., 2010). Mouse models of lupus showed differences in microbiota composition, as compared to control mice. Females showed a more accelerated development of the disease that was associated with decreased levels of *Lactobacillaceae* and increased levels of *Lachnospiraceae* families (Zhang et al., 2014). Treatment with probiotics composed by *Lactobacillaceae* members has been used as anti-inflammatory therapies given its anti-inflammatory properties (Jirillo et al., 2012). Lopez et al. observed the influence of the microbiota over T cell differentiation in healthy and SLE patients (Lopez et al., 2016). These authors found an increase in lymphocyte activation and differentiation toward Th17 in SLE patients (Lopez et al., 2016). Even though, there is still no clarity about the role of microbiota in the development of SLE, the evidence suggests that the dysbiosis observed in the SLE patients could be related to this disease.

### Psoriasis, psoriasis arthritis, and other skin related autoimmune pathologies

The literature provides evidence for an association between intestinal microbiota with skin autoimmune diseases (Scher et al., 2015; Eppinga et al., 2016; Forbes et al., 2016; Zakostelska et al., 2016). Intestinal-related autoimmune diseases like Crohn's disease is a known comorbidity of psoriasis, patients with psoriasis have a 2.9-times higher risk of developing Crohn's disease as compared to the general population as well as Crohn's disease patients have a 7-times higher risk of developing psoriasis (Oliveira Mde et al., 2015). There is increasing evidence supporting a role of intestinal dysbiosis as a factor in the pathogenesis of Crohn's disease (Kaur et al., 2011) and this might as well be related to psoriasis pathology.

Another important comorbidity of psoriasis is psoriatic arthritis, which is a type of chronic spondyloarthritis with unknown etiology. A recent study characterized the composition of gut microbiota in patients with psoriatic arthritis, patients with psoriasis and healthy controls. The gut microbiota profile of both groups showed decreased bacterial diversity and a reduced relative abundance of some bacterial taxa as compared to healthy controls, such as *Akkermansia, Ruminococcus*, and *Pseudobutyrivibrio*. It is important to highlight that the microbiota profile of psoriatic arthritis resembled that published for patients with IBD, a finding that further supports a possible role for the gut microbiota in this skin disease (Scher et al., 2015). The directionality of this relationship remains poorly understood, but it opens a new and interesting research field.

Other skin diseases related to intestinal dysbiosis are scleroderma and vitiligo. In scleroderma (also known as systemic sclerosis) the majority of patients experience gastrointestinal tract dysfunction that might be related to gut microbiota composition. A study performed by Volkmann et al. (2017) shows a different microbial composition between two different cohorts of patients with systemic sclerosis and healthy controls. *Firmicutes* showed a significantly increased abundance in systemic sclerosis patients (63.5 and 42.8%) compared to healthy controls (33%). In contrast, *Bacteroidetes* decreased in one of the cohorts (21.3%) as compared to healthy controls (63.2%). This study also associated the presence of some genus with disease severity. In both systemic sclerosis cohorts, *Clostridium* was more abundant in patients with low gastrointestinal symptom severity, *Lactobacillus* was more abundant in patients with mild constipation and *Prevotella* was more abundant in patients with moderate to severe gastrointestinal symptom severity. Further research will elucidate the role and molecular pathways in which these genders contribute to disease pathogenesis (Volkmann et al., 2017).

Even though, there is information relative to the influence of intestinal microbiota over skin autoimmune diseases, there are data that make these pathologies more complex. Therefore, an influence of skin microbiota over the skin autoimmune diseases remains to be demonstrated (Sanford and Gallo, 2013; Statnikov et al., 2013; Tett et al., 2017). Briefly scientific work that relates skin microbiota with skin autoimmune diseases will be discussed due to the importance of the skin as a physical barrier that constantly interacts with factors from the environment, such as solar irradiation, grades of humidity or dryness and microorganisms. While a wide range of microbes inhabit the skin, the principal residents microbiota belong to one of these phyla: *Actinobacteria, Bacteroidetes, Firmicutes*, or *Proteobacteria*. Interactions between these microorganisms at the skin and the immune system have been suggested to influence tissue integrity and homeostasis (Sanford and Gallo, 2013). Similar to what happens in the gut, skin dysbiosis has been postulated to contribute to the pathology of diverse skin autoimmune diseases (Zeeuwen et al., 2013) like psoriasis, psoriasis arthritis and vitiligo (Gao et al., 2008; Castelino et al., 2014; Ganju et al., 2016). Studies related to chronic plaque psoriasis, the most common form of psoriasis, have shown that the composition of the microbiota both with skin lesions or not differed of healthy skin (Gao et al., 2008; Alekseyenko et al., 2013). Gao et al. showed that *Firmicutes* were the most abundant and diverse phylum present in the psoriatic patients with skin lesions (relative abundance of 46.2%) as compared to those that did not display lesions (39%) and with healthy subjects (24.4%) (Gao et al., 2008). In contrast, *Actinobacteria* has lower relative abundance in psoriatic patients with lesions (37.3%) compared to individuals with healthy skins (47.6%) (Gao et al., 2008). The phylum of *Proteobacteria* was less frequent in psoriatic patients (11.4%) as compared to subjects with healthy skin (21.9%). Alekseyenko et al. described three distinct types of bacteria from their data about the relative abundance of major phyla present in the skin, *Actinobacteria, Firmicutes*, and *Proteobacteria* (Alekseyenko et al., 2013). These results are consistent with some of the findings described above. Proteobacteria dominate the cutaneotype 1 they described, while the cutaneotype 2 has higher relative abundance of *Actinobacteria* and *Firmicutes*. These authors associated the cutaneotype 2 with psoriasis status in which the higher abundance of *Firmicutes* coincides with the results by Gao et al. (2008). The differences in the results could be attributed to as to where the sample was obtained from, as it has been demonstrated that skin microbial composition is dependent on the area of the body that is analyzed (Grice and Segre, 2011). Both studies provide evidence that skin microbiota composition is different in patients with psoriasis as compared to healthy controls. Nevertheless, the physiological meaning of these differences has yet to be defined.

The most recent study to date related to psoriasis is a metagenomic analysis that focused on the less explored diversity of the skin microbiota and provided evidence that these less characterized species inhabiting the skin might be relevant in disease pathogenesis. In this study, an increase in members of the genus *Staphylococcus* was associated to development of psoriasis (Tett et al., 2017).

The skin microbiota is not the only microbial compartment that has been associated to psoriasis pathogenesis. In a murine model of imiquimod-induced psoriasis-like skin inflammation, germ-free, and oral antibiotic-treated mice showed milder skin inflammation as compared to conventional reared mice, along with a decrease in γδTCR and Th17 cells in draining lymph nodes and spleen. These cells are essential components of the IL-23/Th17 axis, which happens to be the main axis in psoriasis pathogenesis. These results suggest that the absence of microbiota or an alteration in their composition caused by antibiotic treatment can decrease the pro-inflammatory T cell response and thus diminish the severity of the imiquimod-induced skin inflammation (Zakostelska et al., 2016). Interestingly, in humans, a link between psoriasis and Crohn's disease has long been acknowledged (Hughes et al., 1983) and discussed extensively in previous articles (Najarian and Gottlieb, 2003). Finally, a recent study characterized for the first time the skin microbial composition of patients with the autoimmune disease vitiligo (Ganju et al., 2016). The data obtained were used to characterize a core skin microbiome for a lesional and non-lesional skin (Ganju et al., 2016). *Methylobacterium* constitutes a genus exclusive to lesional skin while *Anaerococcus, Microbacterium, Streptophyta*, and *Nocardiode* are exclusive to non-lesional skin (Ganju et al., 2016). It remains unclear whether the microbiota differences between groups are a cause or an effect of altered skin physiology of the depigmented patches. Therefore, further research is required to define the contribution of the bacterial component to the pathology of vitiligo (Ganju et al., 2016). Compared to the vast information about the role of microbiota in systemic autoimmune diseases, the contribution of intestinal microbiota to skin disease conditions has not been fully explored and requires further research.

## Autoimmunity and microbiota in psychiatric disorders

Psychiatric disorders have complex etiologies likely resulting from environmental interactions with genetic factors (Tsuang, 2000; Chaste and Leboyer, 2012). Recently, comorbid immune system dysregulations have emerged as a relevant etiological agent in several psychiatric disorders, raising the concept that autoimmunity might be an important contributing factor (Severance et al., 2016). This notion is supported by occurrence of brain inflammation-induced psychosis and autoimmune encephalitis, such as the anti-NMDA receptor encephalitis. This latter disease is characterized by the presence of autoantibodies against the *N*-methyl-*D*-aspartate receptor (NMDAR), a glutamatergic receptor in the brain involved in synaptic transmission (Guasp and Dalmau, 2017). Symptoms include a range of psychotic symptoms early in the course of the disease followed by neurologic deterioration and ultimately protracted cognitive and behavioral deficits (Dalmau et al., 2011). Remarkably, approximately 80% of patients recover with immunotherapy directed to remove the antibodies and antibody-producing plasma cells (Dalmau et al., 2017). Consistently with this notion, various autoantibodies have been detected in subgroups of schizophrenic patients (Pearlman and Najjar, 2014). In particular, meta-analyses have suggested that schizophrenic patients are three times more likely to have high levels of anti-NMDAR antibodies as compared to healthy controls (Pearlman and Najjar, 2014). Recently, Schwarz et al. analyzed fecal microbiota from a small cohort of patients with first-episode psychosis (FEP) (Schwarz et al., 2017). Interestingly, *Lactobacillus* and *Bifidobacterium* were increased in FEP patients and correlated with the psychotic symptoms severity (Schwarz et al., 2017). Although up to date this is the only report associating changes in the microbiota with psychotic severity in patients, it was also reported that autistic children show increased amounts of Lactobacillus in their intestinal microbiota (Adams et al., 2011; Schwarz et al., 2017). At family level, Schwarz et al. observed a decreased in the *Veillonellaceae* family an alteration of the microbiota composition that was also observed in depressive patients (Jiang et al., 2015).

As the largest organ of the immune system, the GI tract serves as an interface between the environment and the host. Therefore, alterations in gut cellular processes may influence immune homeostasis. Data generated to date suggest that increased intestinal permeability and alterations in the gut microbiota composition are strongly associated with psychiatric disorders and could represent potential sources of immune functional impairment (Fiorentino et al., 2016; Esnafoglu et al., 2017; Stevens et al., 2017). Gut microbiota seems to play a critical role in the bidirectional communication between the gastrointestinal tract and the CNS. In addition to regulating gastrointestinal functions, this microbiota-gut-brain axis has been shown to modulate brain functions, such as emotional behavior and stress-related responsiveness (Diaz Heijtz et al., 2011). The mechanisms implicated in these pathways, although not entirely characterized, involve humoral and/or neural route, such as the vagus nerve (De Palma et al., 2014). An involvement of the vagus nerve in the communication between the gut and the brain was supported by the observation that vagotomy abrogated the decrease of anxiety- and depression-related behavior induced by the probiotic *L. rhamnosus* in mice (Bravo et al., 2011). These observations suggest a close relationship between the different components of the gut-brain axis, several probiotics used in animal models have shown their efficacy with combined and correlated beneficial effects on the GI tract, such as intestinal barrier strengthening, HPA axis activation and behavior, including social interaction, anxiety, and exploratory behavior (Hsiao et al., 2013; Vanhaecke et al., 2017). On the other hand, mice exposed to antibiotics to alter the established microbiota, showed an increased in explorative behavior associated with an enhanced level of brain-derived neurotrophic factor (BDNF), an important growth factor for neuronal survival and synaptic plasticity (Bercik et al., 2011). However, another mouse strain exposed to a different antibiotic mixture induced a different behavioral phenotype consisting of an increased depressive-like behavior and altered social interaction, which was associated with reduced level of hippocampal BDNF activity. These data suggest that mouse strains or antimicrobial regimen can differently influence microbiota composition and in turn differently affect behavior (Guida et al., 2017). The use of the probiotic *Lactobacillus casei DG* in these animals was able to restore the behavioral phenotype and BDNF levels similar to controls (Guida et al., 2017). Interestingly, a decreased serum BDNF levels were observed in patients with major depressive disorders (Pedrotti Moreira et al., 2017) and pharmacological treatment that increases BDNF levels proved to be beneficial for depressive patients (Gupta et al., 2016). Along with the depressive-like behavior, mice exposed to antibiotics showed a marked dysbiosis characterized by a loss of bacterial diversity, an increase in *Protebacteria* and *Actinobacteria* and a decrease in *Bacteroidetes* and *Firmicutes*. The probiotic treatment was not able to restore this bacterial phenotype, but promoted the increase of *Lachnospiraceae*, a fiber-degrading and SCFA producer (Guida et al., 2017). In humans, either a decrease or an increase of *Lachnospiraceae* has been correlated with major depressive disorders (Naseribafrouei et al., 2014; Fung et al., 2017), indicating that there is still a controversy about microbiota composition in this pathology. Such a discrepancy could probably result from the influence of external factors (i.e., diet) between the patients cohorts used in these studies. The causal link of microbiota on GI and brain dysfunction has recently been shown for irritable bowel syndrome (IBS), a common intestinal disorder often accompanied by comorbid anxiety (De Palma et al., 2017). Colonization of germ-free mice with fecal microbiota from IBS patients induced altered GI transit, intestinal barrier dysfunctions, and anxiety-like behavior, suggesting a role for the gut microbiota in the expression of IBS (De Palma et al., 2017). The importance of microbiota has also been highlighted in neurodevelopmental disorders, such as autism spectrum disorder (ASD). In mice, pregnant animals exposition to poly(I:C) triggers a maternal immune activation that can cause in the offspring to atypical social and repetitive behavior reminiscent of ASD-related behavior. The mechanisms leading to behavioral abnormalities in the offspring required a particular type of maternal bacterium, the segmented filamentous bacteria (SFB), which induces intestinal T_H_17 cells producing IL-17 (Kim et al., 2017). Interestingly, induction of T_H_17 response by SFB has been shown to modulate gene expression of enteric neurons, which in turn influences microbiome/immune system crosstalk (Yissachar et al., 2017). Besides behavioral abnormalities, offspring from poly(I:C)-treated mice also develop GI symptoms, changes in gut bacterial population and intestinal barrier defects, as reported in some patients with ASD (Horvath and Perman, 2002). Treatment of the offspring with *B. fragilis* induced a remodeling of the microbiota composition ameliorated GI symptoms and reduced repetitive behavior. These preclinical results stimulated the development of pilot clinical studies using microbiome-driven therapies for ASD. Although not controlled and double-blinded, a clinical trial based on microbiota transfer of healthy donors in 7–16 years old children with ASD showed improvements in GI symptoms and in social and communication skills that are associated with bacterial community changes (Kang et al., 2017). Similarly, probiotic supplementation with *Bifidobacteria and Lactobacillus* species in 5–9 years ASD children modified the fecal microbiota composition and improved both GI symptoms and behavior (Shaaban et al., 2017). However, these studies were performed with a small number of patients, and wide-scale randomized controlled trials are needed to critically confirm the efficacy of probiotics in ASD. The use of probiotics or microbiota-derived metabolites that could selectively or simultaneously act on the different components of the microbiota-gut-brain axis raises alternative new treatments for CNS disorders. However, more information is still required to better understand the influence of the microbiota on CNS pathogenesis and to delineate the cellular and molecular mechanisms involved in the communication between microbiota, the gut, and the brain. Also, the implications of intestinal dysbiosis in the etiology and/or pathophysiology of brain diseases remain to be further defined and represent a major challenge for translational research.

## External factors that influence the microbiota

Microbiota is established by many factors that determine the characteristics for each individual, including genetic predisposition, an inheritance from the mother since the fetus is forming until breastfeeding and environmental factors, such as diet, culture, and geographic location (Yatsunenko et al., 2012). However, when there is an imbalance of microorganism's populations present in the gut, known as dysbiosis, many problems at the systemic level are triggered. This situation may cause an incorrect nutrient absorption, favor weight alterations and enhance some immune system diseases (Degruttola et al., 2016).

Diet is one of the important factors affecting the composition of microbiota in humans (Conlon and Bird, 2014; Sonnenburg and Backhed, 2016). The first major change in diet that humans experience is when the consumption of solid food begins. At this stage, microbiota composition and abundance suffer significant modifications, such as a decrease of *Bifidobacteria* and *Enterobacteria* (Fallani et al., 2011). This process occurs due to the new substrates that are available in the gut, leading to the proliferation of certain types of microbes (Fallani et al., 2011; Davis et al., 2017; Wampach et al., 2017). Later in life, each time that diet changes this phenomenon takes place, promoting the growth of some microorganisms more than others (Ottman et al., 2012; Odamaki et al., 2016; Sonnenburg and Backhed, 2016). In turn, this evolving microbiota will modulate the development and function of the immune system (Round and Mazmanian, 2009; Conlon and Bird, 2014; Kabat et al., 2014). However, microbiota analyses highlighted that although after a month of receiving a traditional diet, the composition of the microbiota took 1 year to change and be similar to the one found in other people with the same diet (Claesson et al., 2012).

Human studies have shown that there is a correlation in elderly between the microbiota location and diet, which can influence their health (Claesson et al., 2012). Additionally, analyses of microbiota composition had shown four different dietary groups and found that diet based on “low fat-high fiber” was associated with a more diverse microbiota. A study performed with C57BL/6 mice fed with high-fat diet was aimed at evaluating whether inflammation was due to microbiota changes or cytokines production. Results have shown that microbiota underwent changes 8 weeks after the treatment started, but increased IFNγ and TNF-α cytokines were only detected 16 weeks after administration of a high-fat diet (Guo et al., 2017). These observations underscore the importance of microbiota and corroborate the ability of diet to modulate microbiota composition.

Additionally, evaluation of the feces obtained from three different dietary groups ovo-lacto vegetarian, vegans, and omnivores, showed differences in microbiota composition (Ferrocino et al., 2015). Findings showed that *B. fragilis* group was diminished on ovo-lacto vegetarian and vegan volunteers, which is associated with low consumption of protein and animal fat (Wu et al., 2011). Also, a significant reduction for the loads for Lactic Acid Bacteria (LAB) was observed for the dietary groups ovo-lacto vegetarian and vegan, which may be due to the absence or low intake of some food such as cheese and yogurt (Zimmer et al., 2012).

Gluten-free diet is worldwide used for patients with celiac disease (CD), which is a chronic enteropathy caused mainly by gluten intolerance. It has been shown that the intestinal microbiota can be modified by a gluten-free diet: two of the groups that are reduced by such a diet are *Lactobacillus* and *Bifidobacterium*, which are considered as part of the group of healthy bacteria (De Palma et al., 2009; Lorenzo Pisarello et al., 2015). In contrast, some opportunistic bacteria from *Enterobacteriacea* family are increased as a result of gluten-free diets (De Palma et al., 2009; Lorenzo Pisarello et al., 2015). One possible explanation for these changes in the microbiota is the low carbohydrate intake due to the restrictions imposed by the gluten-free diet. Healthy bacteria usually use carbohydrates for their metabolism, which are needed for the colonization and fermentation inside the gastrointestinal tract (Sanz, 2010).

A diet that low in carbohydrates accessible for microbiota results in low levels of production of Short Chain Fatty Acids (SCFA) produced by bacteria from the microbiota. Butyrate, propionate, and acetate can modulate immune system by controlling inflammation and promoting an anti-inflammatory environment in the gut (Maslowski et al., 2009; Arpaia et al., 2013). However, when the diet undergoes a detrimental change, the abundance of these beneficial bacteria decreases and therefore SCFA production is reduced. Alteration of SCFA is common in obesity, celiac disease, and Type 2 Diabetes, because it is known that their action ameliorates the disease (Lin et al., 2012; Hong et al., 2016; Lerner et al., 2016).

Consistently with this notion, the Ma-Pi2 diet a diet rich in carbohydrates, whole grains and vegetables was shown to ameliorate dysbiosis and increment the diversity of microbiota in Type 2 Diabetes patients, as compared to untreated patients. Also, abundance of SCFA producers increased, such as *Bacteroides, Dorea*, and *Faecalibacterium*. Furthermore, the implementation of this diet has shown a potential role in the recovery of metabolic control in Type 2 Diabetes (Fallucca et al., 2014; Candela et al., 2016).

### Overuse of antibiotic treatments

Overuse of antibiotics may cause a significant imbalance in microbiota and a disruption of the natural interaction between the microorganisms. One of the most important characteristics of a normal microbiota is the capacity to compete out infectious pathogens (Kamada et al., 2013a). Therefore, microbiota removal by antibiotics may allow the detrimental growth of pathogenic bacteria populations, increasing the probability of an infection. Additionally, antibiotics not only kill pathogens but also beneficial bacteria, eliminating as well the positive effect of the latter. Microbiota modulates the immune response through the molecules it produces, so if beneficial microorganisms decrease, a decrease in the modulation of the immune system can also be observed (Langdon et al., 2016).

In response to a constant exposure of an antibiotic, microorganisms can acquire genetic resistance, leading to an the increment of multi-drug resistance microorganisms (Jernberg et al., 2007), which can become a major public health problem due to the lack of new treatments capable of eliminating these bacteria. In the last years, many cases of infection caused by multi-drug resistant bacteria were reported and this number is expected to increase (Karam et al., 2016; Lee et al., 2016). The effects of overuse of antibiotics can be treated but not reversed. Restoration of the microbiota can take months or even years, but it will not be able to become the same as before (Jernberg et al., 2007, 2010). Importantly, it has also been described that newborns whose mothers have received antibiotics perinatally have a different microbiota composition, as compared to newborns whose mother have not been treated (Fallani et al., 2011). Due to the importance of first microorganisms in the gut of newborn, these changes in microbiota caused by excessive use of antibiotics may have long-term consequences (Langdon et al., 2016).

Interestingly, recent findings have highlighted the natural presence of antibiotic resistance genes in microbiota and their differential occurrence according to diet. Microbiota changes observed in ovo-lacto vegetarian and vegan diets, as compared to omnivores diet, are related to the presence of antibiotic resistance genes. A study performed with 144 volunteers found the presence of 12 antibiotic resistance genes in their microbiota. Among these genes, the occurrence of *erm*(A) (Erythromycin resistance methylase gene) has been exclusively detected in the feces of vegan subjects, which also show a low abundance of *tet*(K) (Tetracycline efflux protein), as compared to the genes present in the feces of omnivores. Interestingly, another antibiotic resistance gene with a higher occurrence observed in the feces of omnivores was *van*(B) (Vancomycin resistance gene; Milanovic et al., 2017).

## Conclusions

**Figure 2:**
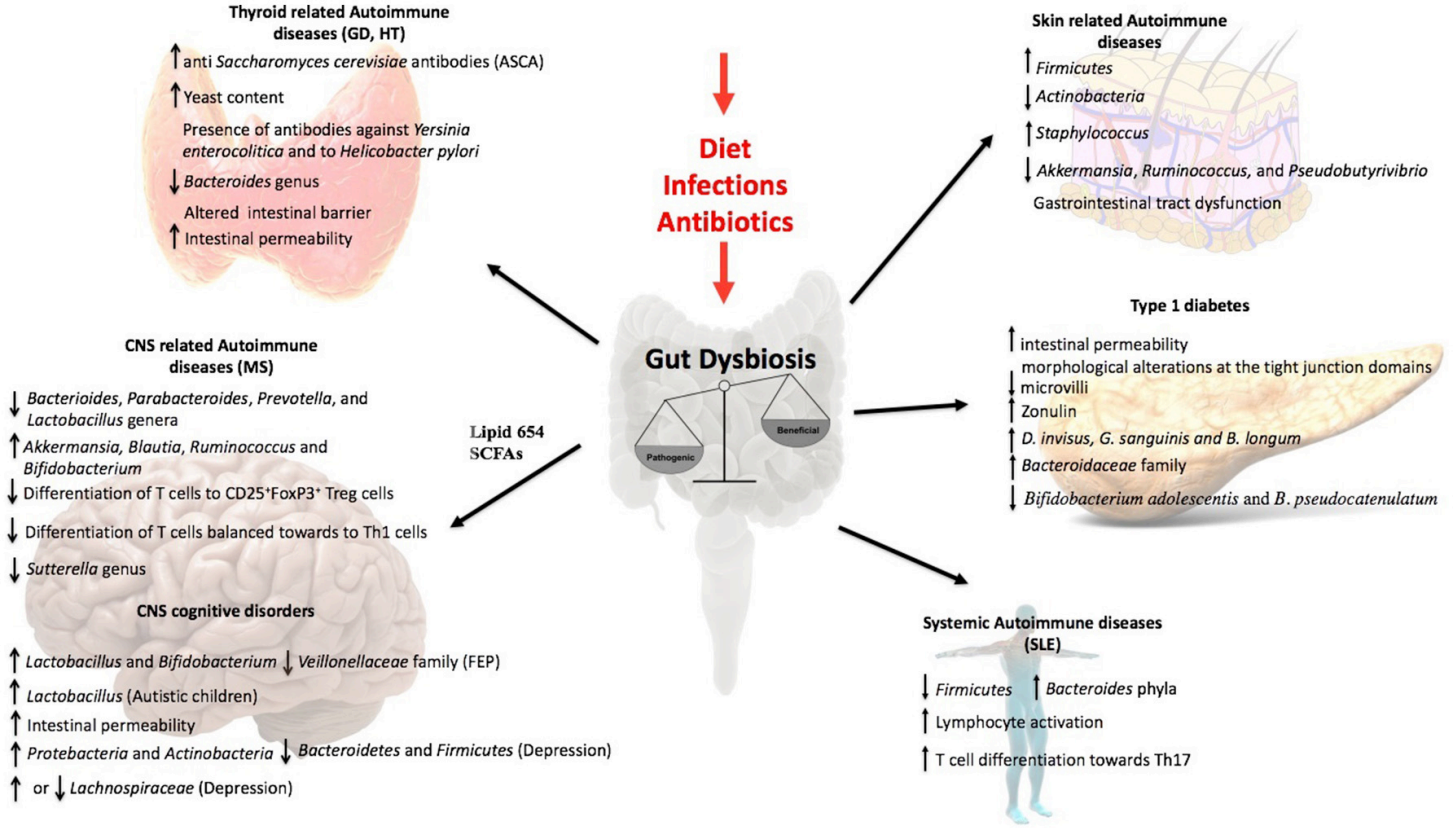
Influence of the gut microbiota in non-intestinal diseases. Gut dysbiosis induced by external factors as diet, infections, or antibiotic overuse lead to an inflammatory response that influence the outcome of several autoimmune diseases as Grave's disease, Hashimoto's Thyroiditis, Multiple Sclerosis, SLE, and type1 diabetes. It has also been observed an important role in skin related autoimmune diseases as Psoriasis. Moreover, the evidences in the literature support that gut microbiota can influence CNS disorders like autism, depression, and schizophrenia.

This review article discusses data supporting the influence of the gut microbiota over non-intestinal autoimmune diseases. The central theme of this review is the intestine in which two important actors, microbiota and the immune system are controlling the response to non-intestinal autoimmune diseases (Figure 2, Table 1). Not much is known about the mechanisms of the interaction between microbiota and the immune system. However, today it is possible to identify certain members of the microbiota that regulate, balance or unbalance the immune response of the host. The current evidence supports the notion that changes or alterations of the microbial species that form part of the intestinal microbiota will affect the balance of Tregs and Th17 cells at the intestine, which could modify the immune response of non-intestinal autoimmune diseases. The experimental evidence suggesting that the cytokines secreted from Treg and Th17 will determine and influence non-intestinal autoimmune responses. It could also be possible that cells of the immune system located at the intestine could to move other organs to establish or modify an autoimmune response. The major message of this review is that the abundant data support the notion that the intestine is a critical organ the appropriate immune balance and for the prevention of non-intestinal autoimmune diseases. The key point is that by modifying the intestinal microbiota of a patient that suffers non-intestinal autoimmune disease it might be possible to improve the outcome of such illness. Interestingly, by modifying the diet it might be possible to improve the intestinal microbiota to promote an anti-inflammatory response of a patient suffering from autoimmunity. Thus, the scientific community has paid attention to the potential therapeutical benefits of manipulating the composition of the gut microbiota through oral administration of probiotic or modified organisms expressing selected self-antigens to treat these non-intestinal autoimmune diseases. Work remains to be done in order to fully understand the complex mechanisms of the intestinal microbiota that can impact non-intestinal autoimmune diseases.

**Table 1:** Alterations observed in the microbiota in non-intestinal autoimmune diseases.

**Autoimmune disease**	**Observation**	**References**
Grave's disease	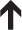 yeast Presence of antibodies against *Y. enterocolitica* and *H. pylori*. 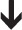 *Bacteroides*	Covelli and Ludgate, 2017 Kohling et al., 2017 Indigo, 2017
Hashimoto's thyroiditis (HT)	Dysbiosis Altered intestinal morphology 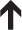 intestinal permeability	Sasso et al., 2004
Multiple Sclerosis (MS) Murine EAE model Human	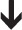 *Sutterella* 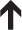 intestinal permeability 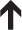 Zonulin expression Th17 > Treg Dysbiosis 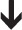 *Bacteroides* 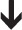 Parbacteroides 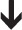 *Prevotella* 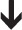 *Lactobacillus* 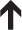 *Akkermansia* 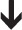 *Blautia* 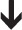 *Ruminococcus* 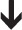 *Bifidiobacterium*	Berer et al., 2017 Nouri et al., 2014 Sturgeon and Fasano, 2016 Nouri et al., 2014 Chen et al., 2016 Jangi et al., 2016 Freedman et al., 2017
Systemic Lupus Erythematosus (SLE) Mouse model of SLE Human	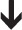 *La ctobacillus* 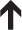 *Lachnospiraceae* Dysbiosis 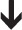 *Firmicutes* 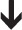 *Bacteroides*	Hevia et al., 2014 Zhang et al., 2014
Psoriasis	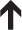 *Firmicutes* 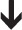 *Actinobacteria* 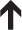 *Proteobacteria* (Cutaneotype 1) 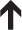 *Actinobacteria* (Cutaneotype 2) 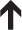 *Firmicutes* (Cutaneotype 3) 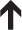 *Staphylococcus*	Gao et al., 2008 Alekseyenko et al., 2013 Tett et al., 2017
Psoriatic arthritis	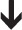 *Akkermansia* 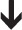 *Ruminococcus* 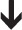 *Pseudobutyrivibrio*	Scher et al., 2015
Scleroderma	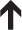 *Firmicutes* 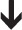 *Bacteroides* 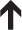 *Clostridium* 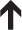 *Lactobacillus* (mild gastrointestinal symptoms) 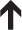 *Prevotella* (moderate to severe gastrointestinal symptoms)	Volkmann et al., 2017
Vitiligo	*Methylobacterium* in lesional skin *Anaerococcus* in non-lesional skin *Microbacterium* in non-lesional skin *Streptophyta* (non-lesional skin) *Nocardiode* (non-lesional skin)	Ganju et al., 2016
Type 1 Diabetes	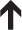 intestinal permeability 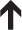 Zonulin 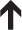 *Bacteroideaceae* 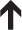 *Blautia* 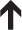 *Rikenellaceae* 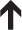 *Ruminococcus* 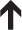 *Sreptococcus*	Secondulfo et al., 2004; Sapone et al., 2006; Maffeis et al., 2016 Sapone et al., 2006 De Goffau et al., 2013 Kostic et al., 2015

## Author contributions

MO, EO-R, and IC-A, have written the first draft of the manuscript. LB, CR, SB, HB, MN, and AK revised and improved the first draft. All authors have seen and agreed on the finally submitted version of the manuscript.

### Conflict of interest statement

The authors declare that the research was conducted in the absence of any commercial or financial relationships that could be construed as a potential conflict of interest.
